# The Neuronal Activation of Deep Cerebellar Nuclei Is Essential for Environmental Enrichment-Induced Post-Stroke Motor Recovery

**DOI:** 10.14336/AD.2018.1220

**Published:** 2019-06-01

**Authors:** Qun Zhang, Jun-fa Wu, Qi-li Shi, Ming-yue Li, Chuan-jie Wang, Xin Wang, Wen-yuan Wang, Yi Wu

**Affiliations:** ^1^Department of Rehabilitation Medicine, Huashan Hospital, Fudan University, Shanghai, China.; ^2^Stem Cell and Regenerative Medicine Laboratory, Ningbo Second Hospital, Zhejiang, China.; ^3^University of Chinese Academy of Sciences, Beijing, China.; ^4^Interdisciplinary Research Center on Biology and Chemistry, Shanghai Institute of Organic Chemistry, Chinese Academy of Sciences, Shanghai, China.; ^5^Department of Rehabilitation Medicine, The Third Affiliated Hospital, Sun Yat-sen University, Guangzhou, China.; ^6^Department of Rehabilitation, Clinical Medical College, Yangzhou University, Jiangsu, China

**Keywords:** DCN, environmental enrichment, Htr2a, photothrombosis, optogenetics

## Abstract

The level of cerebellar activity in stroke patients has been shown to correlate with the extent of functional recovery. We reasoned that the cerebellum may be an important player in post-stroke rehabilitation. Because the neurons in the deep cerebellar nuclei (DCN) represent virtually all of the output from the cerebellum, in this study, using environmental enrichment (EE) to promote rehabilitation, we investigated the influence of the optogenetic neuronal modulation of DCN on EE-induced rehabilitation. We found that neuronal inhibition of the DCN almost completely blocked motor recovery in EE treated mice, but the stroke mice with neuronal activation of the DCN achieved a similar recovery level as those in the EE treated group. No difference was observed in anxiety-like behavior. Moreover, *Htr2a* in the DCN, the gene encoding *5-HT2A* receptor, was shown to be a hub gene in the protein-protein interaction network identified using RNA-seq. This indicated that *5-HT2A* receptor-mediated signaling may be responsible for DCN-dependent functional improvement in EE. We further verified this using the *5-HT2A* receptor antagonist, MDL100907, to inhibit the function of *5-HT2A* receptor in the DCN. This treatment resulted in impaired recovery in EE treated mice, who performed at a level as poor as the stroke-only group. Thus, this work contributes to an understanding of the importance of the DCN activation in EE-induced post-stroke rehabilitation. Attempts to clarify the mechanism of *5-HT2A* receptor-mediated signaling in the DCN may also lead to the creation of a pharmacological mimetic of the benefits of EE-induced rehabilitation.

Stroke is a leading cause of disability worldwide [1]. Numerous studies have attempted to enhance post-stroke functional recovery by modulating the activity of the peri-lesional and contra-lesional stroked areas. However, little clinical success has been made to-date [2]. Since post-stroke deficiencies may occur in both adjacent and remotely connected brain areas [3], gaining control over other connected brain structures may represent new treatment possibilities.

The cerebellum is structurally connected to the sensorimotor cortex via the corticopontocerebellar tract (CPCT). Considerable evidence has implicated the cerebellum as an important node in the brain networks that are altered following supratentorial stroke [4], which results in degeneration of the affected CPCT and increased robustness of the unaffected CPCT. Moreover, a positive correlation has been shown to exist between the activation of the affected cerebellum and the extent of functional recovery in stroke patients [5]. Chronic modulation of activity in the lateral cerebellar nucleus has been found to result in better post-stroke motor recovery in rodents [6]. Taken together, these observations indicate the cerebellum may be involved in the process of post-stroke recovery, and neuromodulation of the deep cerebellar nuclei (DCN), which comprise the sole output pathway of the cerebellum, appears to be a promising approach for stroke rehabilitation. Despite this, the exact role of the DCN in rehabilitation has yet to be extensively described.

Environmental enrichment (EE) is a classical but effective therapeutic intervention for stroke rehabilitation in rodents. In comparison to a standard environment, EE provides comprehensive sensory, motor, cognitive and social stimulation [7]. In recent years, some clinical trials have also suggested that the use of EE in human stroke survivors, such as a mixed rehabilitation unit, may lead to health care cost savings due to its ability to improve better recovery and quality of life [8]. EE has been shown to facilitate post-stroke recovery through different molecular mechanisms in distinct brain areas, but the efficacy of EE as it relates to the cerebellum in stroke rehabilitation remains elusive. As previous studies have indicated that EE leads to an improvement in motor function in both cerebellar injury and Rett syndrome [9], the involvement of the cerebellum in EE-induced post-stroke recovery is deserving of further investigation.

In the present study, we aimed to examine the role of the DCN in EE-induced functional recovery following stroke. Specifically, we investigated whether optogenetic neuronal modulation of the DCN, the sole output nuclei of the cerebellum, influenced the efficacy of EE on post-stroke recovery. We also sought to determine the molecular changes within the DCN after exposure to EE using RNA-seq. The candidate molecules revealed in this study will inform future attempts to create a pharmacological mimetic of EE, with the ultimate goal of improving post-stroke rehabilitation.

## MATERIALS AND METHODS

### Animal preparation

All experimental procedures were approved by the Animal Ethics Committee of the Fudan University (Shanghai, China). Male C57BL/6J mice (8-12 weeks of age, 22-25 g body weight) were used in this study. All mice were housed in a 12:12 h light:dark cycle (with 08:00 to 20:00 as light-on hours), in a room with controlled temperature and humidity. All mice were anesthetized by intraperitoneal injection of 1% pentobarbital (50 mg/kg). During surgery, body temperature was maintained at 37°C with a heating pad.

### Photothrombotic stroke (PT)

PT was induced unilaterally in the sensorimotor cortex. Briefly, anesthetized mice were placed in a stereotaxic device (RWD Life Sciences) with the skull exposed. The entire skull was then covered by an opaque template which exposed a circular region of the left sensorimotor cortex (coordinates: rostral to caudal: 2.5 to 1.5 mm, medial to lateral: 0 to 4 mm, relative to bregma). Immediately after intravenous injection of 0.1% Rose Bengal (0.01 mL/g body weight, dissolved in 0.9% NaCl, Sigma), the sensorimotor cortex was illuminated with a 532 nm green laser beam (50 mw) for 10 min at maximum output.

### Stereotaxic Surgery

#### Virus injection and Optic Fiber Implantation

Anesthetized mice were fixed into a stereotaxic device using ear bars. To prevent any corneal damage, ophthalmic ointment was used. A burr hole was drilled into the right side of the cerebellum, and a glass electrode filled with virus was aimed at the DCN. The intended mouse brain DCN stereotactic coordinates, as previously described [10], were as follows: AP=-6.0 mm from bregma, ML=1.5 mm from the midline, DV=-3.5 mm from the skull surface. 200 nL of rAAV-hsyn-eNPHR3.0-mcherry-WPRE-PA (NPHR gene expression group) or rAAV-hsyn-hChR2-mcherry-WPRE-PA (ChR2 gene expression group) were randomly assigned to be injected into the DCN at a rate of 20 nL/min. After injection, the electrode filled with the virus was left in place for at least 10 min before it was withdrawn, and an optic fiber cannula (200 µm) was then slowly implanted into the injection site. The optical fiber cannula was fixed to the skull with dental cement. The mice were returned to the animal facility to recover for 2 weeks before PT surgery.

#### Stimulation Paradigm

Mice received photostimulation beginning on the 7th day post-PT surgery. Stroked mice were placed in an empty cage to allow for free movement with the laser cable connected to the fiber cannula. One session of stimulation consisted of three 1 min stimulations separated by 3 min rest intervals. All of the mice, including the sham-stimulated stroked mice, received daily (7 days/week) stimulations for 2 weeks post-stroke. In the ChR2 gene expression group, a 473 nm blue laser was controlled by a driver (Thorlabs, DC2200), and mice were stimulated with a laser set to 10 Hz, with 20 msec light pulses. In the NPHR gene expression group, a 594 nm yellow laser was controlled by the same driver as above, whereas mice were stimulated with direct current. The laser power operated with a range of 1.2-1.4 mW, which was measured by a power meter before implantation. Stimulations were performed in the morning between 12:00 and 13:00. Sham stimulation (SS) was conducted without turning on the laser.

The day after the final behavioral test, mice were anesthetized and then transcardially perfused with 4% paraformaldehyde. The regions of virus injection were confirmed under a fluorescence microscope. Data from mice with an absence of ChR2 or NPHR gene expression were excluded from the group.

#### Cannula Implantation

A burr hole was drilled on the right side of the cerebellum. Another two holes were drilled near the implantation point for screw insertion. The cannula was then inserted into the DCN and fixed with dental cement. For subsequent pump installation, a 2 cm long PE50 catheter, filled with sterilized artificial cerebrospinal fluid (aCSF), was inserted into the angle arm of the cannula, and the other end of this catheter was sealed for later pump connection. The sealed end of the catheter was put into the pocket made between the shoulder blades of the mice. Mice were allowed to recover for 14 days before they received PT surgery.

#### Osmotic Pump Installation

Mice received osmotic pump installation on the 7^th^ day post-PT surgery. Osmotic pumps were filled with *MDL*100907 (2.5 mM, 180 µL, Tocris) and kept in sterile distilled water at 37°C overnight before installation [11]. In stroked mice, an incision between the shoulder blades was made and an osmotic pump was connected to the free end of the PE50 catheter, which was left in the pocket in advance. The skin was then tightly stitched with a 5-0 suture thread.

#### Housing conditions

After PT surgery, mice were randomly assigned to one of the following groups: sham group, PT group and PT with EE group (PT+EE). Mice in the sham and PT groups were housed in standard cages (294 × 190 × 130 mm) for 7-21 days after stroke, with five mice per cage. Mice in the PT+EE group were housed in larger cages (545 × 395 × 200 mm), containing various different objects (e.g., chains, runners, ladders, pipelines, boxes, etc.) which were changed every day.

### Behavioral tests

All of the behavioral tests took place between 09:00 and 11:00 and were conducted under the illumination of a 60 W light bulb affixed 2.4 m above each apparatus. Mice were brought to the testing room for acclimation 1 h prior to each test. After completion of the behavioral tests, mice were returned to their respective environments for 60 min, after which they were sacrificed for brain tissue. Each apparatus was cleaned with 75% ethanol after removal of each animal.

#### Accelerating rotarod

**Figure 1.**
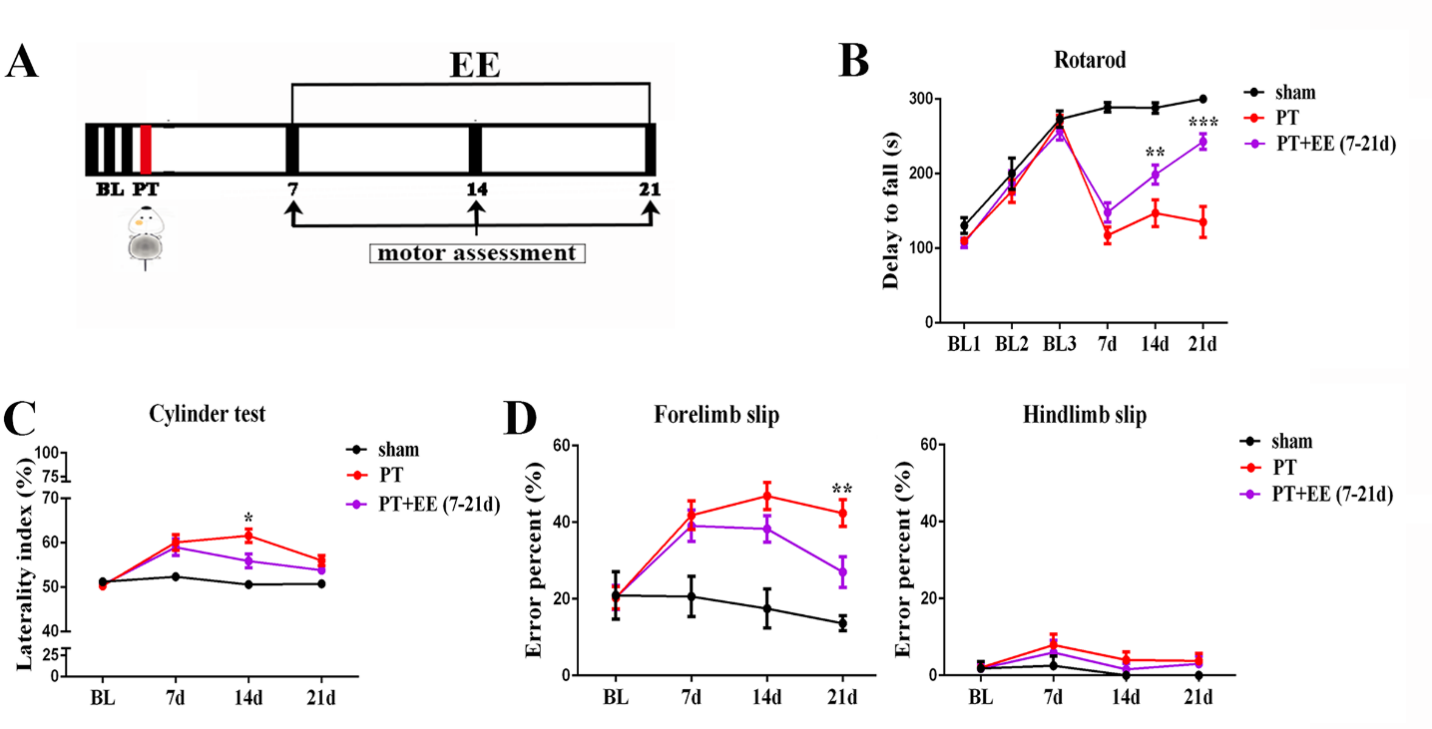
Effects of EE on motor recovery after PT (**A**) Time course of behavioral tests. Motor assessments included the rotarod, cylinder and rung walking tests. BL: baseline data for rotarod. (**B**) Time course of the latency to fall off the rotarod. (**C**) The percentage of laterality in the cylinder test. (**D-E**) The percentage of limb slips in the rung walking test, for both forelimb and hindlimb. Data are expressed as the mean±SEM. ^*^PT vs. PT+EE groups. ^*^p≤0.05; ^**^p≤0.01; ^***^p≤0.001.

The rotarod apparatus (gradual acceleration of 5-40 revolutions per minute [RPM] within 90 s) was used to assess motor performance. The pre-training test (in order to establish a performance baseline), was conducted at a linear accelerating speed of 5-40 RPM for three trials per day for 3 consecutive days. The rotarod test was conducted on the 7th, 14th, and 21st day post-PT surgery, or the 14th day after fiber or cannula implantation (Fig. 1A, 3A, 6B). Each trial lasted a maximum of 5 min, and a 3 min rest interval between trials was allowed to avoid fatigue. The latencies to falling off the rod for each mouse in each trial were recorded, and the average latencies were calculated as the final result. The trial was terminated when mice spun around the rod through three complete revolutions.

#### Cylinder test

Mice were placed in a transparent glass cylinder (diameter 9.5 cm, height 16 cm), with two mirrors angled 45° positioned behind it. The spontaneous rearing and forepaw contacts on the cylinder of each mouse were recorded for 5 min, including contacts with the left paw only, right paw only and both paws simultaneously. The cylinder test was conducted on the 7th, 14th, and 21st day post-PT surgery, or the 14th day after fiber or cannula implantation (Fig. 1A, 3A, 6B). Data were analyzed using asymmetry, which was calculated as follows: (left + 0.5×both) / (right + left + both) ×100%.

#### Rung walking test

The ladder rung apparatus was composed of two plexiglass walls (69.5 cm × 15 cm), which were spaced 5 cm apart to allow for the passage of a mouse but prevented its turning around. A total of 31 plastic bars with a diameter of 0.1 cm were arranged at a distance of 1 cm apart and inserted into the holes on the walls. The entire apparatus was placed on two holders, 17 cm above the ground. Before the PT surgery, mice were trained to cross the ladder for three consecutive trials. The test was conducted on the 7th, 14th, and 21st day post-PT surgery, or the 14th day after fiber or cannula implantation (Fig. 1A, 3A, 6B). With a camera positioned at a slight ventral angle to the apparatus, we were able to record the four limbs of the walking mouse at the same time. The total number of steps and the number of errors made by the right forelimb were then recorded.

#### Elevated plus maze test (EPM)

Anxiety-like behaviors were recorded for 5 min in the EPM. Mice facing one of the closed arms were placed in the central neutral zone at the beginning of the test. The cumulative percentage of time spent in the open and closed arms were subsequently recorded. The test was only conducted on the 21st day post-PT surgery.

### Data analysis

Data from motor behavioral tests were analyzed using a two-way ANOVA with a Tukey’s multiple comparisons post-test. EPM data were analyzed using a one-way ANOVA with a Tukey’s multiple comparisons post-test. An unpaired two-tailed *t-*test was used to analyze qPCR data. All the analysis and statistics were performed using GraphPad Prism Version 6.0. In all cases, a *p* value less than or equal to 0.05 was considered significant. All data are shown as the mean values ± standard error of the mean (SEM).

### RNA-seq library generation and sequencing

Total RNA was isolated from the right DCN using TRizol (Invitrogen, USA) in the PT and PT+EE groups. To generate RNA sequencing libraries, a TruSeq RNA Library Preparation Kit v2 (Illumina) was used, following the manufacturer’s instructions. Pair-end 150 bp sequencing was then performed on a HiSeq x102500 Illumina at the WuXi NextCODE Corporation.

### Quantitative real-time PCR

Total RNA (1 ug) from samples in the PT and PT+EE groups were extracted and converted into cDNA with the Hifair^™^ III 1st Strand cDNA Synthesis SuperMix Kit (YEASEN) in a total volume of 20 µL. Quantitative PCR was performed on the Quantstudio^™^ Real-Time PCR System (Thermo Fisher) in a final reaction volume of 10 μL, using 50 ng of the cDNA template from each sample. GAPDH served as the housekeeping control. Reactions were performed in triplicate with a minimum of three independent runs. Data were analyzed using the ΔΔCt method on Quantstudio^™^ Real-Time (RT) PCR software (Thermo Fisher). Primer sequences are displayed in Table1.

**Table 1:** Primer sequences used for the validation of cerebellum-associated gene expression amongst upregulated genes.

Gene	Forward primer	Reverse primer
Ngb	CTGCTGCCTCTCTTCCAGTA	AGCTGGTCAGGTACTCCTCC
Gabrq	AAATGTGCAGGATGGCCTGA	TGGCCAATTGAGTCTGGCTT
Htr1a	CAGGGCAACAACACCACAAC	CGTTGGAGAGGCCAGTATCG
Htr2a	GCTCTGTGCCGTCTGGATT	CGGCTATGGTGAATGGGGTT
Chrm1	TGCTGGTGCTCATCTCCTTC	CCCATGAGCAGGTATGTGGT

### RNA-seq data processing

The sequencing reads were aligned to the mouse reference genome GRCm38 using TopHat and Bowtie2. Cufflinks were then used to assemble the aligned sequences into transcript isoforms and identify the transcription start sites, using the mouse transcript annotations from Ensembl 81 as a guide. Cuffdiff was used to calculate the FPKM values and test for differential expression. CummeRbund was employed for post-processing of the results in R. The differentially expressed genes were expressed as fold-change with Benjamini-Hochberg FDR corrected q-values <0.05. Hierarchical clustering and heat maps were generated in R.

## RESULTS

### Environmental enrichment promotes motor recovery following photothrombotic stroke

A rotarod test was used to assess motor coordination ability (Fig. 1B). Significant effects of time (*F*[_2, 243_]=52.90, *p*<0.0001), treatment (*F*[_5, 243_]=39.80, *p*<0.0001) and time × treatment interaction (*F*[_10, 243_]=8.84, *p*<0.0001) were observed using a two-way ANOVA test with repeated measures. Upon further analysis with a Tukey’s multiple comparisons test, we found no obvious distinction between groups in the pre-trial training (*p*=0.78). However, compared to the sham group, serious motor deficiencies were observed in the PT group on the 7th day post-PT, with an approximately 60% reduction in the rotarod travel time. As EE started from the 7th day post-stroke onwards, better performance was observed in the PT+EE group compared to the PT group on both the 14th (*p*=0.004) and 21st (*p*<0.0001) day post-PT (n_sham_=9, n_PT_=17, n_PT+EE_=17).

Additionally, the cylinder test (Fig. 1C) and rung walking test (Fig. 1D-E) were conducted, which more sensitively detect impairments in fine motor skills. In the cylinder test, a two-way ANOVA with repeated measures indicated significant effects of time (*F*[_3, 124_]=15.00, *p*<0.0001), treatment (*F*[_2, 124_] =18.60, *p*<0.0001) and time × treatment interaction (*F*[_6, 124_]=3.98, *p*=0.001) among the three groups. On the 7th day post-PT, a marked deficit in the use of the right forelimb and a higher reliance on the contralateral left forelimb was observed in stroked mice of both PT and EE groups. However, on the 14th day post-PT the EE treated group tended to touch the cylinder wall more often with both paws (*p*=0.028), and less often with their left forelimb only (*p*=0.002), in comparison to the PT group. Both PT and PT+EE groups recovered gradually, and by the 21st day post-stroke the two groups displayed equal paw placement times (*p*=0.16; n_sham_=8, n_PT_=13, n_PT+EE_=13).

Similarly, a two-way ANOVA with repeated measures indicated significant effects of time (*F*[_3, 99_]=4.94, *p*=0.003), treatment (*F*[_2, 99_]=14.70, *p*<0.0001) and time × treatment interaction (*F*[_6, 99_]=2.28, *p*=0.04) on forelimb placement among the three groups (Fig. 1D). No significant difference was found in hindlimb slips between the PT and PT+EE groups (Fig. 1E). However, performance in the rung walking test was better in the PT+EE group compared to the PT group. An obvious increase in forelimb slips was observed on the 7th day post-PT. Although no statistically significant difference was found between the PT and PT+EE groups on the 14th day post-PT, a faster and more robust recovery was observed in the EE treated group on the 21st day post-stroke, as assessed by a Tukey’s multiple comparisons test (*p*=0.01; n_sham_=5, n_PT_=10, n_PT+EE_=10).

Despite the fact that the above data collectively suggested a facilitation of motor recovery with EE, the level of improvement in these three behavioral tests appeared to be different; compared to the cylinder and rung walking tests, EE resulted in more robust and faster improvement in the rotarod test.

**Figure 2.**
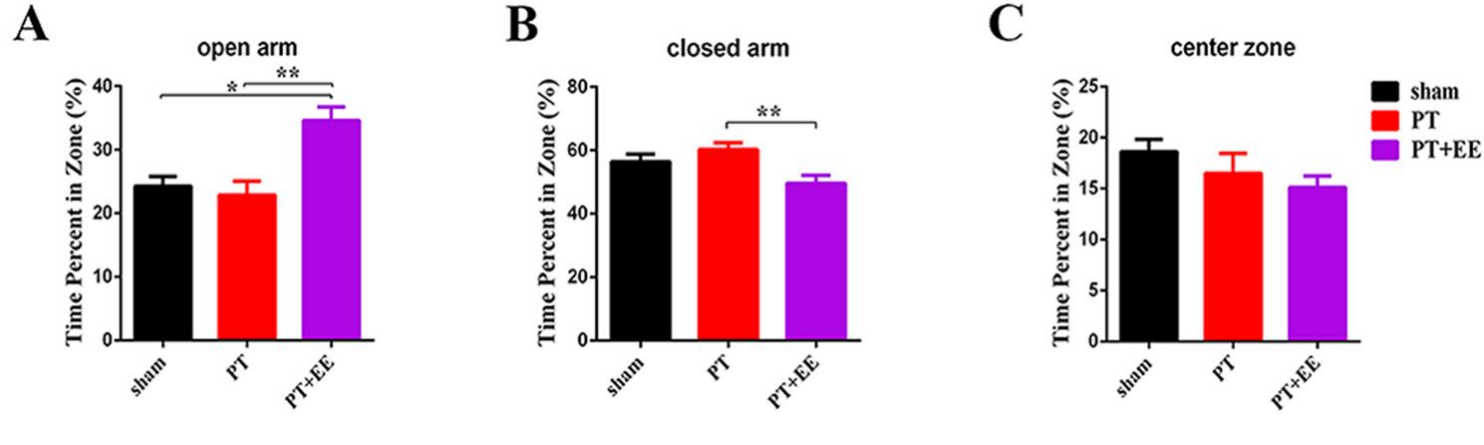
Effects of EE on anxiety-like behavior after PT (**A-C**) The percentage of time mice spent in the open arm (**A**), closed arm (**B**) and center zone (**C**) of the elevated plus maze in each group. Data are expressed as the mean±SEM; ^*^p≤0.05; ^**^p≤0.01.

### Environmental enrichment attenuates anxiety-like behavior

In order to evaluate the exploration ability or anxiety level of mice, we analyzed EPM data by comparing the time spent in each maze zone (Fig. 2). Compared to the PT group, the PT+EE group spent more time in the open arm (Fig. 2A), and less in the closed arm, as assessed by a one-way ANOVA (Fig. 2B; open arm: *F*[_2, 30_]=9.78, *p*=0.001; closed arm: *F*[_2, 30_]=5.84, *p*=0.01). This indicated an attenuating role of EE on anxiety-like behavior. However, as no remarkable differences were observed between PT and sham groups, the PT stroke model appeared to have no effect on anxiety-like behavior. Additionally, the time spent in the center zones (Fig. 2C) was virtually identical among the three groups (*F*[_2, 30_]=0.96, *p*=0.39; n_sham_=7, n_PT_=13, n_PT+EE_=13).

### Repeated optogenetic DCN stimulation modulates environmental enrichment-induced motor recovery

**Figure 3.**
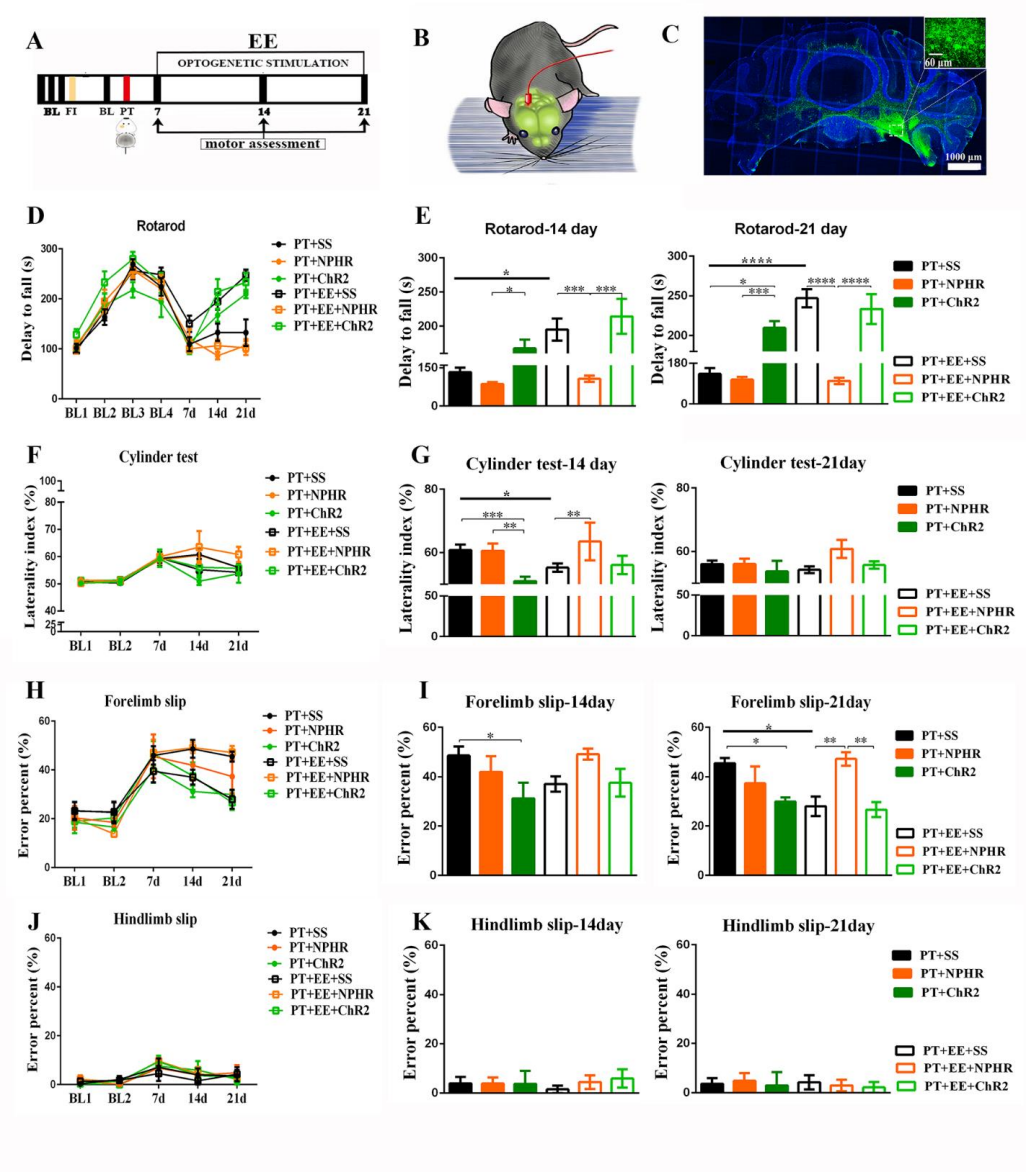
Effects of optogenetic DCN stimulation on motor recovery (**A**) Time course of behavioral tests after optogenetic stimulation of the DCN. Motor assessments included the rotarod, cylinder and rung walking tests. BL: baseline data for rotarod. FI: fiber implantation. (**B**) Schematic depiction of the optogenetic DCN stimulation used to modulate motor function. (**C**) Representative image of viral injection location targeted to the DCN. Positive expression of ChR2-GFP was observed in the DCN (100X magnification, scale bar =1mm). The morphology of the neurons (green) was shown in the inserted image (400X magnification, scale bar = 60μm). (**D**) Time course of the latency to fall off the rotarod in each group. (**E**) Histogram of the falling off latency in each group on the 14th and 21th day after stroke. (**F**) The percentage of laterality in the cylinder test in each group. (**G**) Histogram of the laterality percentage in each group on the 14th and 21th day after stroke. (H and J) The percent of forelimb slips (**H**) and hindlimb slips (**J**) in the rung walking test in each group. (**I, K**) Histogram of the limb slips percentage in each group on the 14th and 21th day after stroke. Data are expressed as the mean±SEM. ^*^p≤0.05; ^**^p≤0.01; ^***^p≤0.001; ^****^ *p*≤0.0001.

To further investigate the role of the DCN in EE-induced recovery after stroke, we used optogenetics to modulate DCN activity, allowing us to simultaneously observe the resulting behavioral changes (Fig. 3B-C).

In the rotarod test (Fig. 3 D-E), significant effects of time (*F*[_6, 293_] =68.60, *p*<0.0001), treatment (*F*[_5, 293_]=11.30, *p*<0.0001) and time × treatment interaction (*F*[_30, 293_]=3.52, *p*<0.0001) were found, as assessed using a two-way ANOVA with repeated measures. Upon further analysis with a Tukey’s multiple comparisons test, we found that, compared to the PT+SS group, repeated DCN activation resulted in an increased latency to fall in the PT+ChR2 group (*p*=0.01) on the 21st day post-PT, which was as long as that of the PT+EE+SS group (*p*=0.56). However, compared to the performance of the PT+EE+SS group (14th day post-PT, *p*=0.001; 21st day post-PT, *p*<0.0001), when the PT+EE+NPHR group was given DCN inhibition, mice exhibited significantly slower and poorer recovery rates, at the same level as those observed in the PT+SS group (14th day post-PT, *p*=0.84; 21st day post-PT, *p*=0.74). No significant difference was found between the PT+SS and PT+NPHR groups (14th day post-PT, *p*=0.32; 21st day post-PT, *p*=0.90; n_PT+SS_=9, n_PT+EE+SS_=10, n_PT+NPHR_=9, n_PT+ChR2_=7, n_PT+EE+NPHR_=8, n_PT+ EE+ChR2_=6).

In the cylinder test (Fig. 3F-G), the effects of time (*F*[_4, 241_]=29.30, *p*<0.0001), treatment (*F*[_5, 241_]=2.74, *p*=0.02) and time × treatment interaction (*F*[_20, 241_]=1.40, *p*=0.12) were also analyzed using a two-way ANOVA with repeated measures. Upon further analysis with a Tukey’s multiple comparisons test, the PT+ChR2 group was found to have a better rate of recovery than the PT+SS group on the 14th day post-PT (*p*=0.001), although not better than that of the PT+EE+SS group (*p*=0.45). However, by the 21st day post-PT, a similar performance was observed in the PT+ChR2 and PT+SS groups (*p*=0.94). Like the observed rotarod behavior, repeated DCN activation in the PT+EE+ChR2 group did not facilitate significant changes in forelimb asymmetry when compared with results from the PT+EE+SS group (14th day post-PT, *p*=0.99; 21st day post-PT, *p*=0.99). Nevertheless, when the PT+EE+NPHR group was treated with DCN inhibition, mice exhibited a robust deterioration in the use of the paralyzed forelimb compared to that of the PT+EE+SS group by the 14th day post-PT (*p*=0.01), which was as poor as that observed in the PT+SS group (14th day post-PT, *p*=0.88; 21st day post-PT, *p*=0.35). Likewise, no significant difference was found between the PT+SS and PT +NPHR groups (14^th^ day post-PT, *p*>0.99; 21st day post-PT, *p*>0.99; n_PT+SS_=13, n_PT+EE+SS_=13, n_PT+NPHR_=13, n_PT+ChR2_=7, n_PT+EE+NPHR_=7, n_PT+ EE+ChR2_=7).

**Figure 4.**
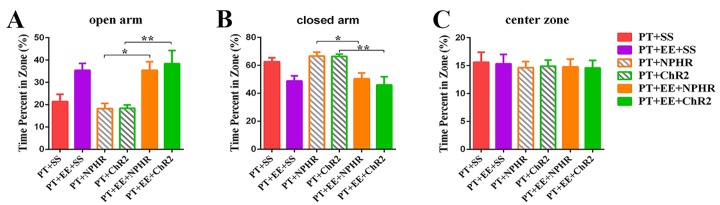
Effects of optogenetic DCN stimulation on anxiety-like behavior after PT The percentage of time spent in the open arm (**A**), closed arm (**B**) and center zone (**C**) of the elevated plus maze. Data are expressed as the mean±SEM; ^*^p≤0.05; ^**^p≤0.01.

In the rung walking test (Fig. 3H-I), the effects of time (*F*[_4, 190_]=46.30, *p*<0.0001), treatment (*F*[_5, 190_]=3.97, *p*=0.002) and time × treatment interaction (*F*[_20, 190_]=1.34, *p*=0.14) were again analyzed using a two-way ANOVA with repeated measures, with further analysis by a Tukey’s multiple comparisons test. Repeated DCN activation resulted in more improvement in the PT+ChR2 group than in the PT+SS group on the 21st day post-PT (*p*=0.02), which was equal to that of the PT+EE+SS group (*p*=0.89). Moreover, repeated DCN activation in the PT+EE+ChR2 group did not facilitate improved recovery relative to the PT+EE+SS group (14th day post-PT, *p*>0.99; 21st day post-PT, *p*>0.99). However, DCN inhibition in the PT+EE+NPHR group resulted in a slower and poorer recovery compared to the PT+EE+SS group on the 21st day post-PT (*p*=0.01), which was identical to that observed in the PT+SS group (*p*=0.99). No significant differences were found in the number of hindlimb slips (Fig. 3J-K) among these groups. (n_PT+SS_=7, n_PT+EE+SS_=10, n_PT+NPHR_=7, n_PT+ChR2_=7, n_PT+EE+NPHR_=7, n_PT+ EE+ChR2_=7).

### Repeated optogenetic DCN stimulation has no effect on the environmental enrichment-induced attenuation of anxiety-like behavior

To identify the potential involvement of the DCN in the EE-induced attenuation of anxiety-like behavior, we again used optogenetics to modulate DCN activity and recorded the time spent in the EPM test (Fig. 4A-C). In line with previous results, we did not observe a significant effect of DCN stimulation, as assessed using a one-way ANOVA, on the amount of time spent in each maze area. The PT+EE groups receiving repeated optogenetic DCN stimulation, either activation or inhibition, all spent significantly more time in the open arm of the maze (Fig. 4A), and decreased time in the closed arm of the maze (Fig. 4B), compared to the PT group (open arm: *F*[_5, 37_]=6.99, *p*=0.0001; closed arm: *F*[_5, 37_]=6.38, *p*=0.0002). There were no significant differences found among all groups in the amount of time spent in the center zone of the maze (Fig. 4C; *F*[_5, 37_]=0.084, *p*=0.99; n_PT+SS_=8, n_PT+EE+SS_=7, n_PT+NPHR_=7, n_PT+ChR2_=7, n_PT+EE+NPHR_=7, n_PT+ EE+ChR2_=7).

#### Htr2a is a hub gene that is expressed differentially in mice exposed to environmental enrichment

**Figure 5.**
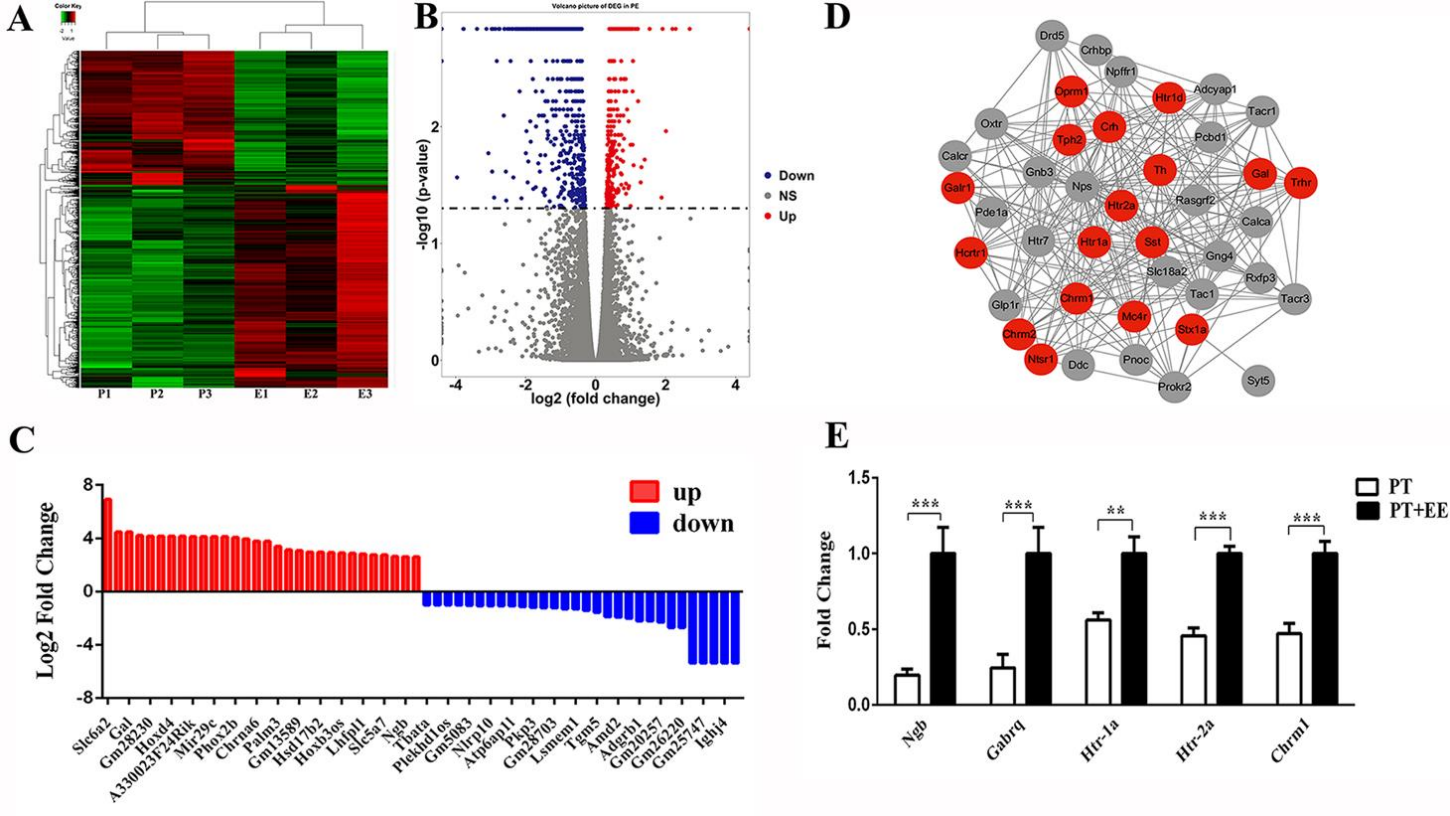
Hierarchical clustering of transcripts in the DCN of PT and PT+EE groups (**A**) Heat map of differentially expressed genes in the PT vs. the PT+EE group. Each group included three independent biological replicates. Red: high, Green: low, log scale from -2 to +1. (**B**) Volcano plots of significant differential expression of mRNAs in the PT versus the PT+EE group. The x-axis represents log_2_-fold change, and the y-axis represents -log10 (*p*-value). Blue dots: genes that were lower in the PT+EE group compared to the PT group. Red dots: genes that were higher in the PT+EE group compared to the PT group. Grey dots: genes that were unchanged in the PT+EE and PT groups. (**C**) Representative distribution of genes in the high expression subset and low expression subset in the PT+EE vs. PT group. Blue column: genes that were lower in the PT+EE group compared to the PT group. Red column: genes that were higher in the PT+EE group compared to the PT group. (**D**) PPI (protein-protein interaction) network of the upregulated genes. The red nodes are known cerebellar target genes. (**E**) RT-PCR validation of the expression of cerebellar-associated genes, from the list of upregulated genes. Data are expressed as the mean fold-change±SEM from three separate experiments, conducted in triplicate. ^**^p≤0.01; ^***^p≤0.001.

To investigate whether the molecular pathways associated with the DCN were altered in mice subjected to EE, we performed RNA-seq on a total of six DCN samples comprising three independent biological replicates from the PT and PT+EE groups. Hierarchical clustering (Fig. 5 A-B) revealed that samples clustered in agreement with the group assignment, with a total of 1,384 differentially expressed genes found between the two groups. The list of genes comprised both annotated mRNAs and unknown transcripts. The top 30 upregulated and downregulated mRNAs in each group are displayed in Figure 5C. Compared to the downregulated genes in the PT+EE group relative to the PT group (n=557), which ranged from 1.23 to 40.90-fold, more robust changes were observed amongst the upregulated genes (n=809), which ranged from 1.23 to 120.26-fold over the PT group. Therefore, we used the top 200 genes from the upregulated list for further protein-protein interaction (PPI) network construction. Strikingly, we found that 41.5% (17/41) of genes in the most connected module were related to normal functioning of the cerebellum, which are denoted as red dots in Figure 5D. Notably, *Htr2a*, which encodes *5-HT_2A_* receptor, a sub-type of serotonin receptors responsible for motor coordination in the cerebellum, tended to be a hub gene in the PPI network. To validate the RNA-seq data, we further tested the selected mRNAs from the list of upregulated genes against the same RNA as that from RNA-seq experiments, which showed consistent changes with the RNA-seq data (Fig. 5E). Together, this experiment identified molecular changes related to DCN function in EE treated samples, allowing us to select *Htr2a* for further experimental examination.

**Figure 6.**
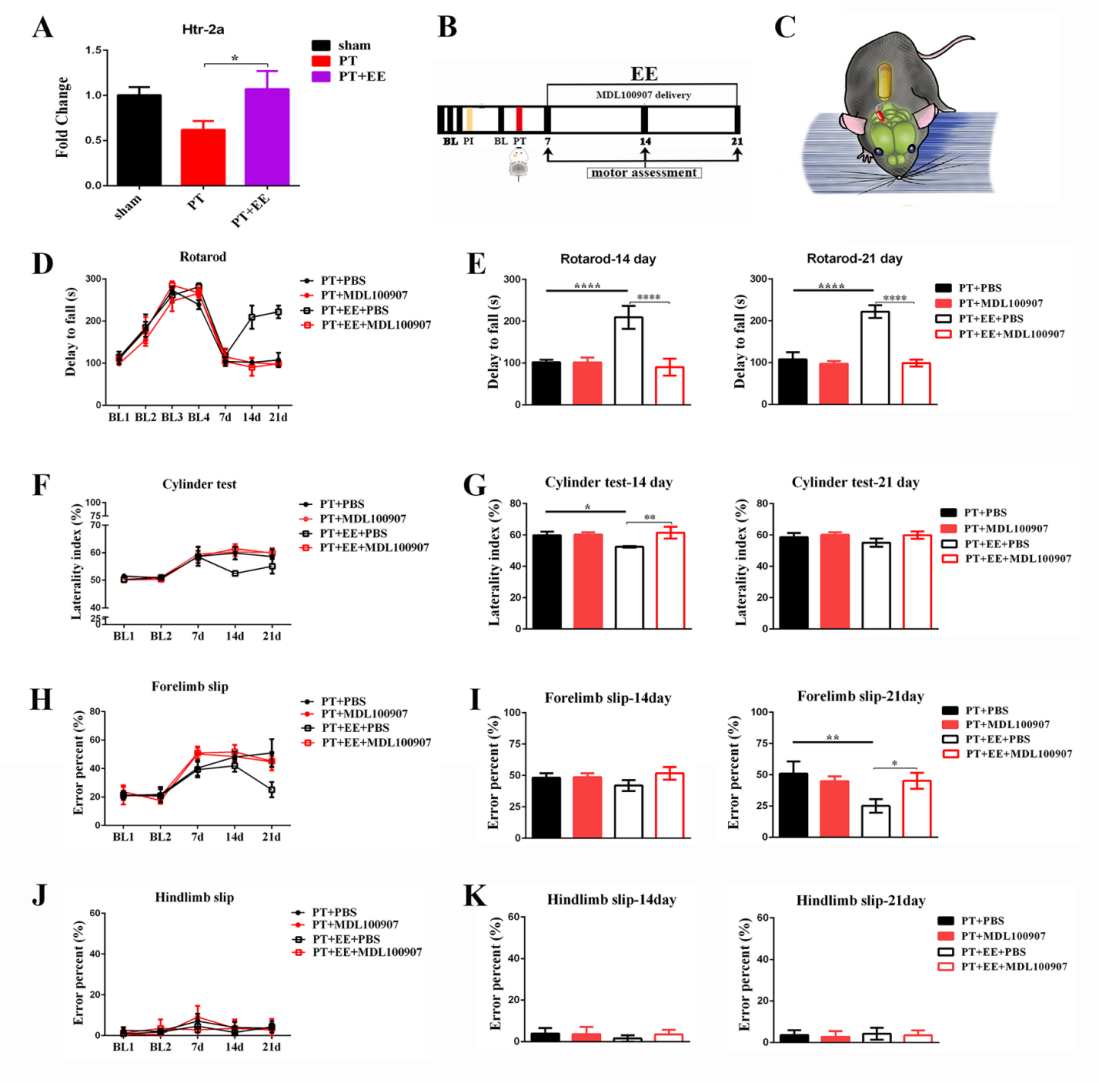
Effects of the *5-HT_2A_* receptor antagonist *MDL*100907 on motor recovery after PT (**A**) Relative expression of *Htr2a* mRNA in each group. (**B**) Time course of behavioral tests after *MDL*100907 administration to the DCN. Motor assessments included the rotarod, cylinder and rung walking tests. BL: baseline data for rotarod. PI: osmotic pump implantation. (**C**) Schematic depiction of the mini osmotic pump implantation filled with *MDL*100907. (**D**) The latency to fall off the rotarod in each group. (**E**) Histogram of the falling off latency in each group on the 14th and 21th day after stroke. (**F**) The percentage of laterality in cylinder test. (**G**) Histogram of the laterality percentage in each group on the 14th and 21th day after stroke. (**H, J**) The percentage of the forelimb (**H**) and hindlimb (**J**) slips in the rung walking test. (**I, K**) Histogram of the error slips percentage in each group on the 14th and 21^th^ day after stroke. ^*^p≤0.05; ^**^p≤0.01, ^****^p≤0.0001.

Next, we examined the expression levels of *Htr2a* in new DCN samples from sham, PT and PT+EE groups (five samples with three replicates in each group) via RT-PCR. Using a two-tailed *t-*test, a significantly (~50%) higher *Htr2a* expression was observed in the PT+EE group compared to the PT group (Fig. 6 A; *t*=2.22, *df*=26, *p*=0.04), whereas no difference was observed between the sham and PT+EE groups (*t*=0.57, *df*=23, *p*=0.57).

### The 5-HT_2A_ receptor antagonist *MDL*100907 inhibits environmental enrichment-induced motor recovery following stroke

We next made use of *MDL*100907, a selective *5-HT_2A_* receptor antagonist, in order to inhibit the receptor’s activity in the affected DCN, and further examine the role of the DCN *5-HT_2A_* receptor in EE-induced recovery after stroke (Fig. 6C).

In the rotarod test (Fig. 6D-E), significant effects of time (*F*[_6, 133_] =77.70, *p*<0.0001), treatment (*F*[_3, 133_]=10.80, *p*<0.0001) and time × treatment interaction (*F*[_18, 133_]=3.04, *p*=0.0001) were observed using a two-way ANOVA with repeated measures. Further analysis with a Tukey’s multiple comparisons test indicated that, compared to the PT+EE+PBS group, the latencies to fall were considerably decreased in the PT+PBS, PT+*MDL*100907 and PT+EE+*MDL*100907 groups. Even given the presence of EE treatment, no improvement in performance was observed in the PT+EE+*MDL*100907 group compared with the PT+PBS group on both the 14th (*p*=0.95) and 21st (*p*=0.98) days post-stroke. Performance of the PT+EE+*MDL*100907 group was significantly worse than the PT+EE+PBS group (14th day post-PT, *p*<0.0001; 21st day post-PT, *p*<0.0001). No differences were found between the PT+PBS and PT+*MDL*100907 groups (*p*=0.96; n_PT+PBS_=8, n_PT+EE+PBS_=5, n_PT+PBS+_ *_MDL_*_100907_=5, n_PT+EE+_*_MDL_*_100907_=5).

**Figure 7.**
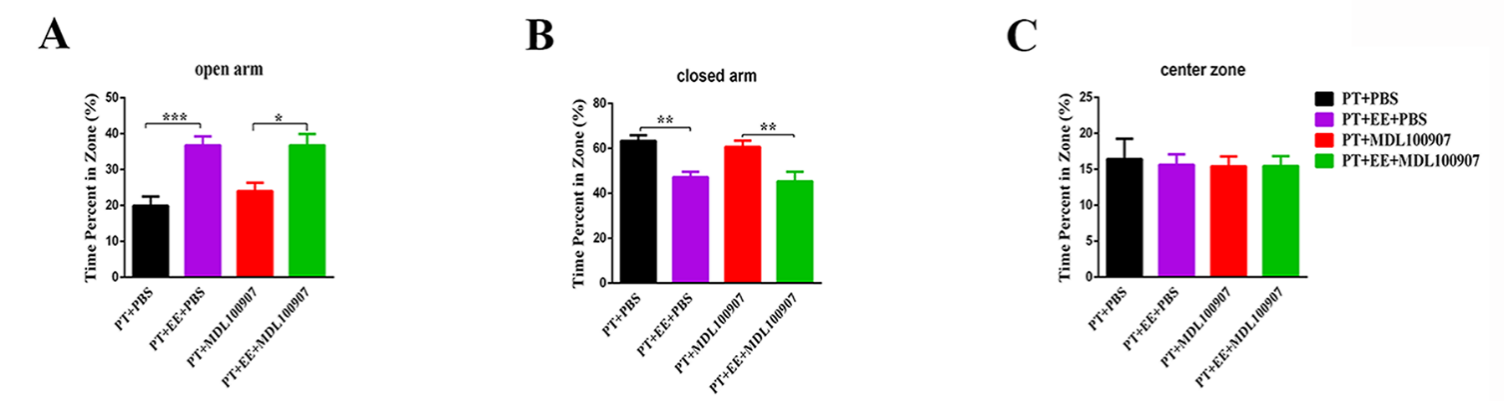
Effect of the *5-HT_2A_* receptor antagonist *MDL*100907 on anxiety-like behavior after PT The percentage of time mice spent in the open arm (**A**), closed arm (**B**) and center zone (**C**) of the elevated plus maze. Data are expressed as the mean±SEM; ^*^p≤0.05; ^**^p≤0.01; ^***^p≤0.001.

In the cylinder test (Fig. 6F-G). The effects of time (*F*[_4, 100_]=26.10, *p*<0.0001), treatment (*F*[_3, 100_]=3.87, *p*=0.01) and time × treatment interaction (*F*_[12, 100]_=0.20, *p*=0.61) were analyzed using a two-way ANOVA. Results definitively demonstrated an inhibition of EE-induced recovery by *MDL*100907. Compared to the PT+EE+PBS group, mice in the PT+EE+*MDL*100907 group tended to rely more on their left forelimb (*p*=0.001) on the 14th day post-stroke. Nevertheless, *MDL*100907 seemed to have no effect on PT, as the PT+*MDL*100907 and PT+PBS groups exhibited similar performance in limb asymmetry (14th day post-PT, *p*=0.99; 21st day post-PT, *p*=0.99). By the 21st day post-stroke, all of the groups exhibited similar forelimb asymmetry (n_PT+PBS_=5, n_PT+PBS+_*_MDL_*_100907_ =7, n_PT+EE+PBS_=7, n_PT+EE+_*_MDL_*_100907_=5).

In the rung walking test (Fig. 6H-I), a two-way ANOVA with repeated measures revealed the effects of time (*F*[_4, 100_]=27.40, *p*<0.0001), treatment (*F*[_3, 100_]=3.01, *p*=0.03) and time × treatment interaction (*F*[_12, 100_]=1.26, *p*=0.26) on forelimb placement among the three groups. No significant differences were found in the number of hindlimb slips (Fig. 6J-K). Although the number of forelimb step errors gradually decreased over time in the PT+EE+PBS group, a Tukey’s multiple comparisons test revealed that the PT+EE+*MDL*100907 group still had a significant number of forelimb slips on the 21st day post-stroke (*p*=0.04), meaning *MDL*100907 was able to block the effect of EE on this behavior test. Since no statistically significant difference was found between the PT+PBS and PT+PBS+*MDL*100907 groups (*p*=0.99), we may conclude that *MDL*100907 administration had no influence on the natural time-course of PT recovery (n_PT+PBS_=6, n_PT+PBS+_*_MDL_*_100907_=7, n_PT+EE+PBS_=6, n_PT+EE+_ *_MDL_*_100907_=5).

### The 5-HT_2A_ receptor antagonist *MDL*100907 has no effect on the environmental enrichment-induced attenuation of anxiety-like behavior

Because the *5-HT_2A_* receptor is involved in motor recovery, we further sought to examine whether it plays a role in the EE-induced attenuation of anxiety-like behavior in the EPM test (Fig. 7). A one-way ANOVA analysis indicated that with the post-stroke administration of either PBS or *MDL*100907 (PT+EE+PBS and PT+EE+*MDL*100907 groups, respectively), mice spent more time in the open arm (Fig. 7A), and decreased time in the closed arm of the maze (Fig. 7B), compared to the PT group (open arm: *F*[_3, 29_]=11.24, *p*<0.0001; closed arm: *F*[_3, 29_]=9.07, *p*=0.0002). Among all groups, we found no significant differences in the amount of time spent in the center zones of the maze (Fig. 7C; *F*[_3, 29_]=0.06, *p*=0.98;. n_PT+PBS_=9, n_PT+PBS+_*_MDL_*_100907_=8, n_PT+EE+PBS_=8, n_PT+EE+_ *_MDL_*_100907_=8).

## DISCUSSION

In the present study, we sought to reveal the contributions of the DCN to EE-induced post-stroke rehabilitation. We demonstrated that the ability of EE to promote post-stroke motor function was definitively dependent on the DCN activation. Furthermore, *Htr2a* in the DCN, the gene encoding *5-HT2A* receptor, was identified as a hub gene in our PPI network. This indicates that *5-HT2A* receptor-mediated signaling in the DCN may be necessary for the ability of EE to enhance motor function post-stroke. This work furthers our understanding of EE-induced recovery following stroke and emphasizes the appreciable role of the DCN in the process of rehabilitation.

In our work, EE resulted in the greatest enhancement in the rotarod test, which is a reliable assessment of cerebellum-dependent motor coordination (Fig. 1), compared to the cylinder or rung walking test. This disparity in the degree of improvement among different behavioral tests has led us to propose that the cerebellum may be the brain area most susceptible to EE after stroke. Until now, EE has been found to promote post-stroke plasticity in different brain regions [12, 13]. The role of the cerebellum, however, has been poorly characterized [14]. Given that all outputs from the cerebellum originate from the DCN, a better understanding of the DCN’s involvement in EE may help to promote more effective rehabilitation.

Interestingly, in contrast to previous studies using electrical or optogenetic stimulation of the lateral cerebellar nucleus to improve motor recovery [6, 15, 16], DCN inhibition almost completely blocked motor recovery in EE treated mice, who performed at a level as poor as the stroke-only group. These suggest that EE-induced motor recovery after stroke was dependent on the cerebellar output pathway, and in turn, that the lack of DCN activation was likely the main cause for poor responsivity to EE in stroke animals. The level of cerebellar activity or metabolism has been shown to correlate with the extent of functional recovery in stroke patients [4, 5, 17, 18], our observation of poor outcomes in EE-treated mice with DCN inhibition further emphasizes the importance of post-stroke rehabilitative therapies that may modulate the DCN activation.

Furthermore, our data provides further evidence for the DCN engagement in cortical functional plasticity. As we know, the cerebellum is structurally connected to the sensorimotor cortex, and the activity and excitability of the cerebral cortex is dependent upon ascending input originating from the contralateral DCN [19]. Previous studies have demonstrated that cerebellar activation increases the plasticity of the ipsi-lesional sensorimotor cortex [15, 16, 20]. It is therefore not surprising that we observed better performance in the cylinder and rung walking tests, two sensorimotor cortex-dependent behavioral tests, in the PT+EE group as well as the PT+ChR2 group. We should note, however, that DCN activation in EE-treated mice appeared to result in better recovery rates in the rotarod test, with less improvement in the cylinder and rung walking tests (Fig. 3). This may indicate that the DCN modulation in the cortex-dependent behavioral tests was limited. More precisely, despite normal cerebellar activity being indispensable for the EE-mediated promotion of motor recovery, DCN activation may result in better motor coordination, with further improvements in fine motor ability requiring additional interventions. Future studies will seek to investigate the effects of DCN modulation on structural plasticity after EE, including observing CPCT reconstruction by MRI and exploring the specific molecular changes underlying experience-dependent synaptic plasticity. Moreover, as far as we know, the DCN contain many neuronal subtypes, our stimulation may activate different neuronal subtypes. In the future, we will examine the different neuronal types using specific cell markers and further investigate the effect of optogenetic stimulation of selective neuronal subtypes on post-stroke recovery.

Serotonin (5-HT) has been reported to engage in a variety of physiological functions, including affective responses and cognitive processing [21]. In this study, an RNA-seq analysis revealed the hub gene, *Htr2a*, a gene encoding the serotonin receptor subtype *5-HT2A* receptor, to be differentially expressed in the DCN in post-stroke animals receiving EE. Furthermore, we found that *5-HT2A* receptor-mediated signaling was responsible for EE-induced functional recovery (Fig. 5D). Unexpectedly, an injection of the selective *5-HT2A* receptor antagonist *MDL*100907 into the DCN only blocked EE-induced motor recovery (Fig. 6), without affecting anxiety-like behavior (Fig. 7). Indeed, considerable evidence from rodent studies has implicated the *5-HT2A* receptor in behavioral changes. Unlike other 5-HT receptor subtype knockout mice [22, 23], *5-HT2A* receptor-deficient mice showed more severe motor dysfunction [24-26]. Because our work has revealed an activation of the DCN after EE, as well as an ability of *MDL*100907 to completely block EE-induced motor recovery, we speculate that *5-HT2A* receptor-mediated signaling most likely engages in the DCN activity. This hypothesis may be supported by the observation that *5-HT2A* receptors are widely distributed and expressed in neuronal cell somata and dendrites of the DCN, and 5-HT could regulate the release of many neurotransmitters such as glutamate and GABA in cerebellum [27, 28], but the exact relationship between DCN activation and *5-HT2A* receptor-mediated signaling in the EE-induced post-stroke recovery needs further exploration. Thus, our future studies will employ *5-HT2A* receptor-specific transgenic mice to determine the precise role of *5-HT2A* receptor-expressed neurons in DCN, which may become a promising target for therapeutic modulation of cerebellar function.

In summary, we show here that the DCN activation is indispensable for EE-induced motor promotion after stroke, without being involved in the EE-induced attenuation of anxiety-like behavior. Additionally, we found that *5-HT2A* receptor-mediated signaling may be responsible for DCN-dependent functional improvement in EE. These results suggest that future therapeutic approaches that aim to modulate the DCN activation may be crucial for effective rehabilitation. Moreover, further attempts to clarify the role of *5-HT2A* receptor-mediated signaling in the DCN may lead to the creation of a pharmacological mimetic of the benefits of EE-induced post-stroke rehabilitation.
